# Electrode Choices in Cochlear Incomplete Partition Type III: The Experience of a Romanian Tertiary Unit

**DOI:** 10.3390/biomedicines14071597

**Published:** 2026-07-16

**Authors:** Dan-Cristian Gheorghe, Mihai Dumitru, Gabriela Musat, Adina Zamfir-Chiru-Anton

**Affiliations:** 1 ENT Department, Emergency Hospital for Children “Marie Curie”, Carol Davila University of Medicine and Pharmacy, 041451 Bucharest, Romania; dan.gheorghe@umfcd.ro; 2 ENT Department, Bucharest University Emergency Hospital, Carol Davila University of Medicine and Pharmacy, 050098 Bucharest, Romania; 3 ENT Department, Saint Mary Clinical Hospital, Carol Davila University of Medicine and Pharmacy, 011172 Bucharest, Romania; gabimusat@yahoo.com; 4 ENT Department, Emergency Hospital for Children “Gr. Alexandrescu”, Carol Davila University of Medicine and Pharmacy, 011743 Bucharest, Romania; zamfiradina@yahoo.com

**Keywords:** cochlear implantation, incomplete partition type III, electrode array selection, perimodiolar vs. lateral wall electrodes, internal auditory canal misplacement

## Abstract

**Objectives:** Incomplete partition type III is a rare malformation of the inner ear characterized by the absence of the modiolus, fixation of the stapes, and significant communication between the cochlea and the internal auditory canal (IAC). During cochlear implant (CI) surgery, there is a risk that the electrode array may enter the IAC, resulting in non-specific stimulation of auditory neurons or other nerve fibers inside the IAC. In this study, we present our experience with CI surgery in patients with incomplete partition type III and report the types of devices utilized. **Methods:** A retrospective analysis was conducted on all our patients with cochlear incomplete partition type III who underwent surgical cochlear implantation in a tertiary referral unit over the past 16 years. **Results:** Of the perimodiolar electrodes used initially, four were correctly positioned within the cochlea from initial insertion, and no reinsertion was necessary. A lateral wall electrode array CI was selected to replace the last failed perimodiolar insertion, but it also required two insertion attempts due to its initial misplacement into the internal auditory canal. **Conclusions:** In this study, electrode array selection was influenced by surgeon preference and by intraoperative factors such as the severity of the gusher. Our observations suggest that perimodiolar CIs can be used in selected cases of incomplete partition type III and did not appear to increase the risk of IAC misplacement compared with the lateral wall array used in one patient. However, these findings should be interpreted cautiously as observations from the authors’ experience rather than as generalized conclusions regarding the relative safety of perimodiolar versus lateral wall electrode arrays. Lateral wall arrays may also be misplaced into the IAC, and their use may be considered in cases with severe and difficult-to-control gusher.

## 1. Introduction

Cochlear incomplete partition type III (IP 3) is one of the rarest malformations of the inner ear [1] and was first described by Nance in 1971 [2]. Early clinical and surgical reports pointed to a stapes footplate fixation followed by a gusher after stapedectomy attempts. The disease was reported in both the male and female sexes, although some published studies have named the condition X-linked deafness [3]. This malformation was described in detail by Sennaroglu from a histological point of view. The disease is defined by a lack of bony modiolus and the septum at the base of the cochlea [4]. No abnormalities were described for the posterior labyrinth. Other authors added that a small cochlea and possible reduced dimensions of the semicircular canals (SSCs) can be found in these cases [5]. High-resolution CT scan imaging (HRCT) is required to diagnose this condition. The findings include a bulbous internal auditory canal (IAC), incomplete separation of the cochlea coils from the IAC, and some abnormalities of the first two parts of the intratemporal nerve canal [6,7]. Sennaroglu showed that interscalar septa are present in these cases. Genetic investigations of these patients can demonstrate an association with mutations in the POU3F4 gene [8].

Affected patients present with progressive mixed hearing loss. Increased perilymphatic pressure is considered the pathogenic mechanism for deafness progression [9].

There are significant challenges in the management of these patients. Traditionally, cochlear implant surgery has been the preferred treatment modality. However, recently published data indicate that hybrid (bone–air) stimulation can achieve comparable communication outcomes in certain patients [10].

Risks associated with surgery for this condition include cerebrospinal fluid (CSF) gusher and possible aberrant insertion of the electrode array into the internal auditory canal due to a lack of separation between them [11]. A proper intraoperative method for CI surgery is required to verify the position of the electrodes inserted into the cochlea. This can be achieved through intraoperative fluoroscopy, cochlear view X-ray, computed tomography (CT), and cone beam CT (CBCT) [12,13,14].

The likelihood of electrode migration in the IAC is significantly increased in these cases [1,13]. Revision surgery can be performed in these patients but is always accompanied by risks, such as trauma to the internal auditory canal and facial nerves during electrode removal [15]. There are conflicting reports about the type of electrode arrays needed to prevent initial intracochlear misplacement. Published papers have noted varying approaches, with some authors advocating the use of lateral wall electrodes and others suggesting perimodiolar electrode arrays [5,16,17]. Some surgeons have used both types, and some prefer stiffening of the electrode by keeping the existing stylet inside the array [18].

The cochlea opening must be completely sealed after electrode insertion in these patients due to possible development of meningitis if CSF leak persists. Some cases may require subtotal petrosectomy if infection cannot be prevented [9].

Audiologic results after CI surgery in patients with IP type III vary widely. Some authors reported lower auditory performance and higher levels of stimulation needed compared to patients without IEM [19]. The different types of *POU3F4* mutations could account for the observed variations [20]. Still, many studies report IP type III CI results as similar to those of children without inner ear malformations [5,21,22].

We present our experience with cochlear implantation in children with IP type III, focusing on electrode array selection and intraoperative strategies to prevent complications related to this inner ear malformation. We hypothesize that electrode directionality and stiffness, rather than nominal electrode class (perimodiolar vs. lateral wall), are the critical factors preventing IAC misplacement in IP type III. This study group includes real cases of misinsertions with both types, which are rarely shown with intraoperative imaging. This study analyzes electrode choice, insertion behavior, and intraoperative verification strategies in pediatric IP type III cochlear implantation.

Only five suitable communications addressing cochlear implantation in IP type III during the past decade have been identified in the English literature, with the number of cases varying from one to ten. From these articles, only lateral wall electrode arrays were utilized, with the exception of one publication that employed Advanced Contour from Cochlear, but with the stylet in place, transforming it into a lateral wall electrode array [1,16,18,19,23].

The objective of our study is to present surgical cochlear implantation results using true perimodiolar electrode arrays and to inform all cochlear implant surgeons that any type of electrode design can be employed in these surgeries. The surgical risks associated with IP type III are likely to be primarily related to the inner ear malformation itself and may be less influenced by the type of electrode array used.

## 2. Patients and Methods

Five patients (5 ears) with incomplete partition type III were submitted to cochlear implant surgery in our hospital between January 2009 and December 2025. All patients were male and presented with bilateral profound sensorineural or mixed hearing loss. Diagnosis was established in all cases by HRCT, and MR imaging was used to verify the presence of the cochlear nerves in each case. Four patients received Cochlear Nucleus perimodiolar electrode array implants (532 or 632) (Cochlear Limited, University Avenue, Macquarie University, NSW 2109, Sydney, Australia), and 1 received a Medel lateral wall implant (Flex 24) (MED-EL Elektromedizinische Geräte GmbH, Fürstenweg 77a, 6020 Innsbruck, Austria) after failure of a perimodiolar array insertion.

**Figure 1:**
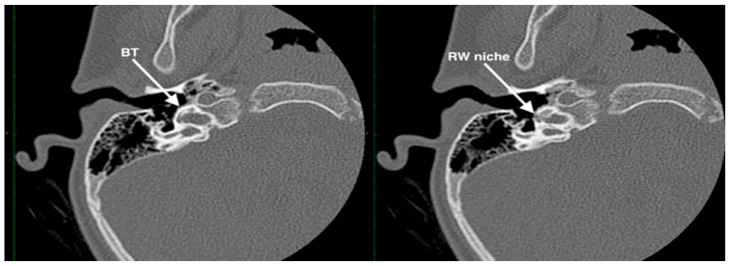
CT scan showing abnormal appearance of the promontory, with difficult interpretation of the middle ear landmarks and the basal turn intraoperatively—patient 1 (RW—round window; BT—basal turn of the cochlea).

**Figure 2:**
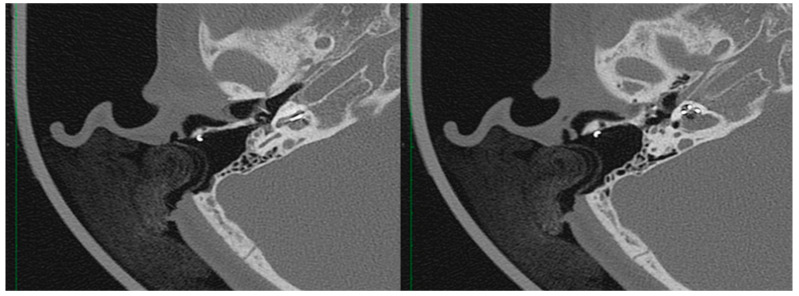
Intraoperative CT scan images of patient 4.

The cochlear implantation was performed by the same surgeon and team. Intraoperative impedance telemetry was performed for each case. Beginning in May 2024, the SmartNav system from Cochlear (Cochlear Americas, 10350 Park Meadows Dr., Lone Tree, CO 80124, USA was utilized to ensure the proper insertion and placement of the electrodes. During the surgical procedure, a computed tomography (CT) scan was performed intraoperatively. The patients were sent to the imaging department to confirm the proper positioning of the electrode arrays within the cochlea. A thick wound dressing was applied to prevent contamination during transportation (see Figure 1). Electrode array reinsertion was necessary in only one case, and a cochlear view X-ray was performed to mitigate further radiation exposure to the patient. The surgical technique employed included a retroauricular approach, mastoidectomy, and posterior tympanotomy. Following the exposure of the round window (RW) niche, the bony overhang was removed if it obstructed the view. The difficulty in recognizing the RW was a consistent issue in all cases. Careful assessment of the middle ear landmarks was performed intraoperatively (and compared with CT scan images) and allowed correct identification of the RW (Figure 2).

In most cases, we prefer to open the round window membrane (excluding the bony contour) on its antero-inferior border using a diamond drill (0.8 mm diameter) at a lower rotational speed to achieve a clear round insertion hole. More rarely, we use the needle to perform the membrane opening. To reduce the CSF efflux, we elevate the head of the operating table, instruct the anesthetist to lower the intracranial pressure, and wait. During this time, we insert the electrode array while fine suctioning is performed around the RW opening. Muscle and fascia pieces are subsequently used to seal the cochlear opening after insertion. If no visible CSF leak persists (observation is carried out for a few minutes and repeated suctioning is performed inside the middle ear space to verify proper sealing), the patient is referred to the radiology department for a CT scan. If the electrode array’s position is deemed normal, an audiology team performs intraoperative electrical ABR testing.

Cochlear implant activation is performed at 4–6 weeks postoperatively. Infant-Toddler Meaningful Auditory Integration (IT-MAIS), Categories of Auditory Performance (CAP), and free-field audiometry with visual reinforcement testing are used in our audiology department to monitor the implanted patients 3, 6, and 12 months after surgery.

This study was conducted in accordance with the Declaration of Helsinki and was approved by the Ethical Committee of our Hospital, Carol Davila University of Medicine (no. 18338, approved on 8 March 2026).

## 3. Results

**Figure 3:**
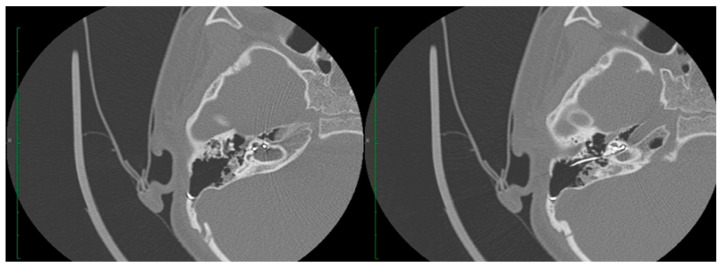
Perimodiolar electrode array position, patient 3.

All cases but one (case 5) received an implant through a single insertion maneuver and perimodiolar arrays (see Figure 2 and Figure 3).

**Figure 4:**
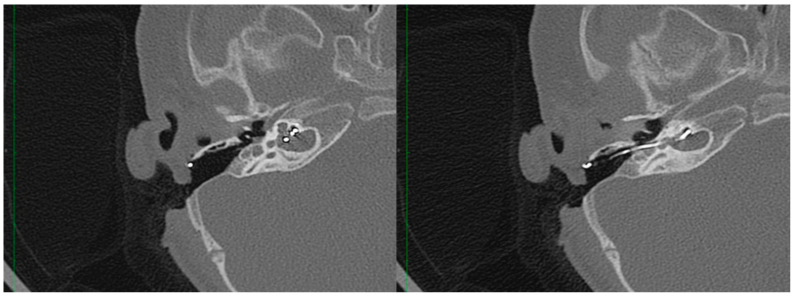
Intraoperative CT scan image from patient 5. The electrode array is located partially in the basal turn but also in the fundus of the IAC.

The last patient (patient 5) demonstrated abnormal insertion of the array, occupying the basal turn entirely, with a slight lean afterwards to the internal auditory canal (IAC) (Figure 4).

**Figure 5:**
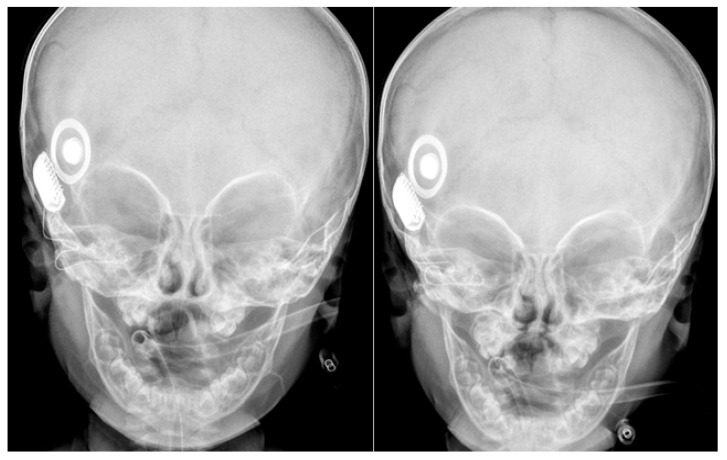
Intraoperative cochlear view X-ray in patient 5. A lateral wall electrode array misplaced inside the IAC (**left**). A second insertion led to correct placement (**right**).

Another attempt was performed at the same position of the electrodes. Due to a particularly severe CSF leak despite thorough sealing of the RW with fascia and muscle, we decided to replace the initial implant (Cochlear 632) with another one having a thicker base electrode array (Medel Flex 24). The initial insertion went smoothly, but the cochlear view X-ray imaging showed an IAC position of the electrodes and a very small portion inside the basal turn (Figure 5, left).

A second attempt using the lateral wall array led to a favorable result, as demonstrated by the intraoperative cochlear view X-ray (Figure 5, right).

In four cases, the CSF gusher was successfully controlled by soft tissue sealing of the cochlear opening in the RW niche. However, in one case, a thicker electrode array was required to control the gusher, in conjunction with the filling of the opening around the electrode entry point with fascia and muscle fragments.

No immediate or late postoperative complications were recorded during the study period. Although the last case was followed up with only 10 months after CI surgery, no local problems related to the implant were noticed, and no signs of possible gusher were present.

**Figure 6:**
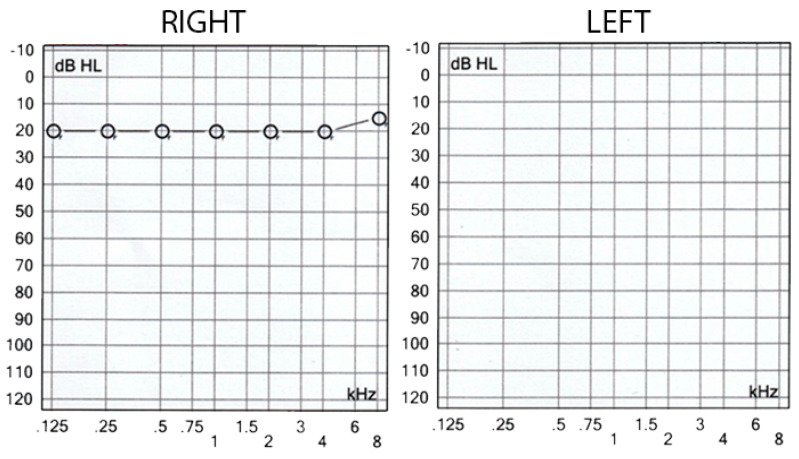
Audiogram of patient 2 (10 years after CI surgery).

**Figure 7:**
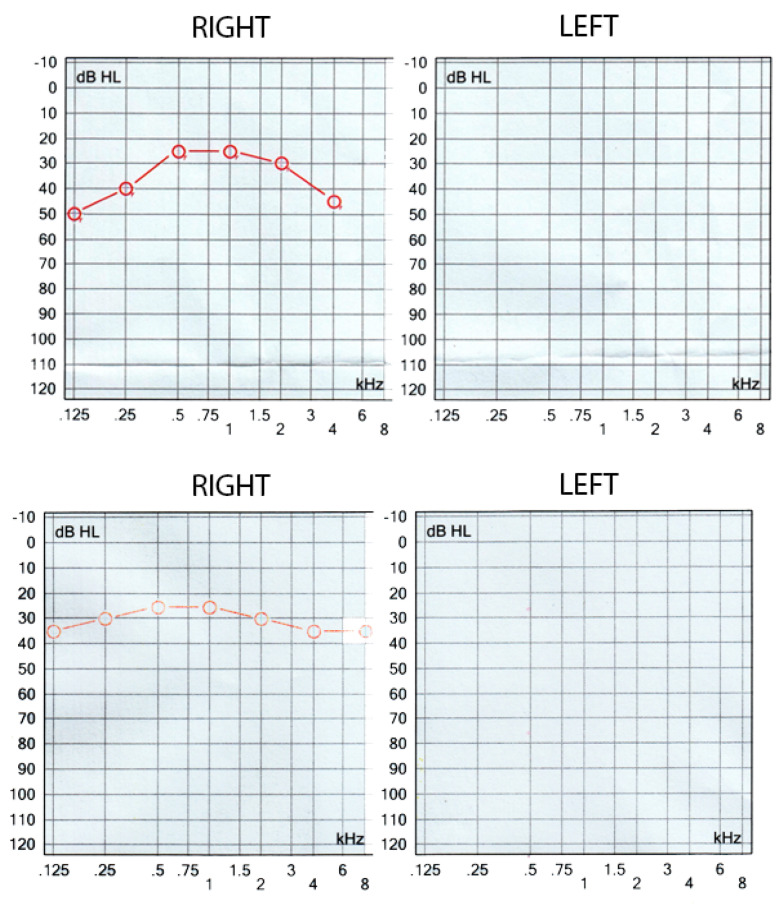
Patient 5 (lateral wall electrode) evaluated with a free-field visual reinforcement audiogram 4 and 9 months after CI surgery.

Presenting auditory data for patients operated on in our department is beyond the intended goals of this paper. All of our CI patients had their devices activated 6 weeks after surgery and were followed up with by our audiology department for at least 2 years. Audiometry, CAP, IT-MAIS, and free-field VRA are techniques used to evaluate our CI patients. To elucidate the auditory outcomes in our IP type III cases in relation to operative trauma, we present the audiograms from two distinct cases: one from a patient with a perimodiolar electrode array (patient 2) and one with a lateral wall electrode array (patient 5). Both demonstrate good auditory thresholds (although at different follow-up times after the CI surgery) (Figure 6 and Figure 7).

Table 1 summarizes the surgical characteristics and outcomes of cochlear implantation in patients with incomplete partition type III.

## 4. Discussion

Due to the absence of a modiolus and anatomical hard separation between the cochlea and the internal auditory canal (IAC), the problem in these patients is likely misplacement of the electrode array. Various reports have documented cases of such issues [15,24]. Low auditory benefits can result due to the reduced contact surface between the electrodes and the cochlear neurons or fibers. Additionally, electrode misplacement during cochlear implantation (CI) surgery can lead to facial nerve or vestibular stimulation. Several methods have been employed to provide feedback on the correct placement of the electrode array within the cochlea. Intraoperative three-dimensional volume tomography (3D-DVT), fluoroscopic guidance, and C-arm beam generators have all been recognized as effective intraoperative techniques to prevent electrode insertion into the internal auditory canal (IAC) [9,14,25], but none of these methods guarantee proper electrode insertion or stability [5].

Our surgical technique involves creating a narrow opening of the RW membrane (0.8 mm) using a low-rotation-speed diamond drill at its antero-inferior border. In all IP type III cases in our study, the cochlear bone is thin, and an extended round window approach is unnecessary because gusher begins the moment we open the inner ear spaces. This minuscule area allows us to insert the electrode sheath from a perimodiolar array inside the cochlea. The sheath aids in lowering the CSF flow and provides us with time to insert the rest of the electrode to its final position. We consider the 3D anatomy of the external osseous cochlea wall when advancing the tip of the electrode array towards the proper direction. This can be achieved by using stiffer and/or perimodiolar electrodes, as they have a dedicated direction of egress from their sheath.

The rotation position and tip direction of the electrode array can be easily controlled using the white triangular support at its base. Consequently, the surgeon has complete control over the direction in which the electrode array is advanced during insertion into an open space, such as that found in the cochlea of patients with IP type III. This technique has been used in four cases, and the proper position of the electrodes (verified by intraoperative CT scan imaging) was ensured based on the first electrode insertion.

A lateral wall electrode array was only used for the case in which a proper insertion could not be achieved, and the gusher could not be stopped by muscle/fascia placed at the RW opening after using a perimodiolar electrode. CT scan imaging was used intraoperatively as in all our previous cases (see Figure 4). The position of the inserted electrodes was in the basal turn, but there was no proper deployment along the upper cochlear turns. Persistence of CSF leakage led us to change the device in favor of one with a thicker base electrode array (Medel Flex24) that could assist in controlling the gusher. Its insertion was not straightforward, since most of it also went inside the IAC, as shown in Figure 5. It first went inside the basal turn and then bent and directed its tip towards the IAC, as seen in the intraoperative cochlear view X-ray. The intraoperative examination protocol was modified to mitigate the risk of administering an elevated dosage of radiation to our patient, particularly in instances where confirmation of insertion was important. A second electrode insertion was eventually achieved. Based on this single case, we cautiously hypothesize that additional anatomical factors in IP type III, not detectable with available imaging techniques, may contribute to difficult electrode positioning inside the cochlea. This interpretation remains speculative and should not be regarded as proof that a specific occult abnormality was present or that electrode design is irrelevant. In the final case in our study (patient 5), the contour of the external wall of the cochlea did not appear to direct the lateral electrode array towards its normal position. The electrode tips from both the perimodiolar and lateral wall arrays failed to enter the upper turns of the cochlea during their respective first attempts, as demonstrated by intraoperative imaging. One possible explanation is that subtle anatomic variations inside the cochlea may have influenced insertion behavior; however, this remains a hypothesis based on intraoperative observation only. These observations suggest that electrode properties such as directionality and stiffness may be relevant, but they should be interpreted with caution given the small number of cases. A perimodiolar electrode array offers both of these properties, and we hypothesize that the sheath of some perimodiolar arrays may help advance and protect the tip of the electrode array until it reaches the lateral wall opposite the point of entrance in the basal turn of the cochlea. Advanced Contour from Cochlear, Standard from Medel, and Mid Scala from Advanced Bionics also share characteristics that could make them candidates for insertion in IP type III cases. Very flexible electrode arrays could be less controllable at insertion. Abnormal insertions have also been reported in the literature, possibly due to the use of a soft and thin electrode array coupled with an abnormal angle of insertion at the RW opening [26]. Other possible abnormalities inside the inner ear may account for misplaced insertions, as seen in our last presented case, but this explanation remains speculative and requires confirmation in larger series or imaging-based studies.

The neurovascular damage risk in reinsertions of electrode arrays has been discussed [27]. Achieving optimal electrode positioning relative to the cochlear external septa appears to be a worthwhile objective, even in the presence of cochlear trauma from reinsertion. The last case in our study showed that despite cochlear trauma from multiple reinsertions, good auditory responses were recorded two months after cochlear implant (CI) activation. Audiogram evaluation revealed a PTA of 31.25 dB (as shown in Figure 7), suggesting minimal intraoperative auditory nerve damage at the time of CI surgery.

Some difficulties regarding the associated gusher persist when using perimodiolar electrodes. Some authors have designed special electrode arrays (featuring a cork stopper at the cochlear entrance) to prevent this complication [1]. Other authors recommend subtotal petrosectomy in these cases and blind sac closure of the external auditory canal [24]. In four out of five cases, we encountered no difficulties in managing the condition. However, this situation presents a clear dilemma when selecting a specific type of cochlear implant. It is imperative to address this issue during preoperative discussions with the parents of the child to adequately prepare them to decide on a particular brand or manufacturer of cochlear implants.

Some papers report on achieving larger cochlea spaces through extended RW drilling [16]. The idea is to obtain a good visualization of the basal turn of the cochlea [28]. We do not favor this approach due to the higher risk of persistent postoperative cerebrospinal fluid (CSF) leak. There is also a certain overlap of terminology describing the procedure itself. Preservation of the RW edges is crucial to maintain the possibility of local retention and fixation of fascial or muscular fragments. Instead, in a normal cochlea, we prefer drilling inside the tympanic scala, just inferior to the RW, to obtain a clear view of the lateral wall of the basal turn. This drilling process is not necessary in IP type III and can exacerbate the gusher. We prefer a 0.8 mm opening of the RW on its antero-inferior margin. We did not observe any residual gusher after cochlear implant surgery in patients with IP type III. Additionally, no meningitis was recorded during the follow-up period of our patients. We did not use a postoperative lumbar drain in any of these cases and ensured that the sealing of the inner ear spaces was completed intraoperatively, and no leakage could be elicited at the end of surgery.

The angle of insertion may be particularly important for lateral wall electrodes, as a flexible tip could be more susceptible to drifting towards unintended locations. Possible explanations include the absence of rigid boundaries between cochlear scalae and the continuous pulsations associated with gusher flow during insertion. These mechanisms are proposed as hypotheses only and should be interpreted cautiously, as they were not directly demonstrated in the present study. Consequently, a stiffer electrode array, including a modiolar-hugging type, may represent a useful option in selected cases, but this possibility requires further confirmation. Notably, the sheath of newer perimodiolar electrode arrays circumvents the base of the cochlea and positions the electrode tip at the lateral wall of the basal turn at the commencement of insertion.

Auditory results after CI in patients with incomplete partition type III are similar to those of individuals without inner ear malformations [5]. We observed similar outcomes when using perimodiolar electrode arrays. Certain authors proposed that employing a full-band electrode array could theoretically enhance the stimulation of auditory nerve fibers [14]. This technique could also help control intraoperative gusher, but the dimensions of the array may cause more trauma to the cochlear nerve fibers. There could also be a higher risk of facial nerve stimulation with full-band electrode arrays due to excitation spreading [28].

There are some limitations that must be acknowledged. Our study is a retrospective one, and the small number of cases only allows us to propose a hypothesis based on our surgical experience. With an increase in diagnosed cases, we can follow this type of IEM and its management. Our electrode selection protocol introduces a specific bias that enables patients to select a device or an alternative, while the surgeon can confirm the compatibility of the electrode array in cases of inner ear malformations. Both perimodiolar CI devices and lateral wall devices receive a comparable level of reimbursement. Our hospital also benefits from having a wide range of manufacturers’ products available at all times, which provides us with the flexibility to adapt to challenging cases and surgical situations. Long-term monitoring of our patients with cochlear implants may also lead us to draw more reliable conclusions in the future regarding the types of electrode arrays the surgeon can use and the effect of intraoperative multiple insertions on auditory preservation in IP type III. The LEESPQ test has recently undergone validation for Romanian children. It has been introduced to our Audiology Department and is likely to become a standard tool for monitoring our Cochlear Implant (CI) population in the future [29].

## 5. Conclusions

Cochlear implantation in children with incomplete partition type III presents a complex surgical challenge. In this study, electrode array selection, whether lateral wall or perimodiolar, reflects the surgeon’s preference and the intraoperative severity of the gusher. Based on our surgical experience, electrode arrays with sufficient stiffness and controllable tip directionality may help guide insertion toward the cochlear turns. In our limited experience, perimodiolar arrays were not associated with a higher frequency of IAC misplacement than the lateral wall array; however, this observation should not be interpreted as evidence of comparative safety between electrode designs. The lateral wall array used in one patient also initially entered the IAC before successful reinsertion, suggesting that misplacement may occur with either design in IP type III. Therefore, these findings should be regarded as a single-center experience rather than generalized conclusions, and larger studies are needed to clarify the relative safety and optimal selection of electrode arrays in this malformation.

## Figures and Tables

**Table 1:** Summary of cases.

Patient No./Name	Sex	Age	Implanted Ear	Electrode Array Type	Manufacturer/Model	Number of Insertion Attempts	Initial Misplacement	Intraoperative Imaging Used	Final Electrode Position	CSF Gusher Control
1. C.T.	M	8y	RE	Perimodiolar	Cochlear Nucleus CI532	1	No	Intraoperative CT	Correct intracochlear position	Controlled with RW sealing
2. C.S.A.	M	6y	RE	Perimodiolar	Cochlear Nucleus CI632	1	No	Intraoperative CT	Correct intracochlear position	Controlled with RW sealing
3. D.I.C.	M	4y	RE	Perimodiolar	Cochlear Nucleus CI632	1	No	Intraoperative CT	Correct intracochlear position	Controlled with RW sealing
4. R.A.	M	5y	RE	Perimodiolar	Cochlear Nucleus CI532	1	No	Intraoperative CT	Correct intracochlear position	Controlled with RW sealing
5. M.L.	M	1y	RE	Lateral wall	MED-EL Flex 24	2	Yes—IAC	Cochlear view X-ray	Correct cochlear placement after reinsertion	Controlled with thicker array + RW sealing

## Data Availability

All data are available from the corresponding author upon reasonable request.

## Abbreviations

The following abbreviations are used in this manuscript:

3D DVT	Three-dimensional digital volume tomography
ABR	Auditory brainstem response
CAP	Categories of auditory performance
CBCT	Cone beam computed tomography
CI	Cochlear implant
CSF	Cerebrospinal fluid
CT	Computed tomography
HRCT	High-resolution computed tomography
IAC	Internal auditory canal
IEM	Inner ear malformation(s)
IP type III/IP III	Incomplete partition type III
IT MAIS	Infant Toddler Meaningful Auditory Integration Scale
MED EL	Medical Electronics (company name, often retained as brand)
MR/MRI	Magnetic resonance/magnetic resonance imaging
POU3F4	POU class 3 homeobox 4 gene
PTA	Pure tone average
RW	Round window
SSCs	Semicircular canals
